# Large eddy simulation of turbidity currents in a narrow channel with different obstacle configurations

**DOI:** 10.1038/s41598-020-68830-5

**Published:** 2020-07-30

**Authors:** Danial Goodarzi, Kaveh Sookhak Lari, Ehsan Khavasi, Soroush Abolfathi

**Affiliations:** 1 Department of Civil, Water and Environmental Engineering, Shahid Beheshti University, Tehran, Iran; 2 CSIRO Land and Water, Private Bag No. 5, Wembley, WA 6913 Australia; 3 School of Engineering, Edith Cowan University, 270 Joondalup Drive, Joondalup, WA 6027 Australia; 4 Department of Mechanical Engineering, University of Zanjan, Zanjan, Iran; 5 School of Engineering, The University of Warwick, Coventry, CV4 7AL UK

**Keywords:** Civil engineering, Applied mathematics

## Abstract

Turbidity currents are frequently observed in natural and man-made environments, with the potential of adversely impacting the performance and functionality of hydraulic structures through sedimentation and reduction in storage capacity and an increased erosion. Construction of obstacles upstream of hydraulic structures is a common method of tackling adverse effects of turbidity currents. This paper numerically investigates the impacts of obstacle’s height and geometrical shape on the settling of sediments and hydrodynamics of turbidity currents in a narrow channel. A robust numerical model based on LES method was developed and successfully validated against physical modelling measurements. This study modelled the effects of discretization of particles size distribution on sediment deposition and propagation in the channel. Two obstacles geometry including rectangle and triangle were studied with varying heights of 0.06, 0.10 and 0.15 m. The results show that increasing the obstacle height will reduce the magnitude of dense current velocity and sediment transport in narrow channels. It was also observed that the rectangular obstacles have more pronounced effects on obstructing the flow of turbidity current, leading to an increase in the sediment deposition and mitigating the impacts of turbidity currents.

## Introduction

Density currents, also known as gravity currents, are primarily horizontally moving fluid flow with higher densities than ambient flow, as a result of variations in temperature and concentration of dissolved and suspended particles^1^. In particular for the latter, currents with varying concentrations of suspended particles are also referred to as turbidity currents^2^. In turbidity currents, buoyancy-driven forces propagate the denser fluid into the ambient fluid with lower density. The suspension and deposition of sediments resulted by turbidity currents could limit the level of functionality and effectiveness of hydraulic structures by reduction in the storage capacity and an increased chance of erosion^3^. Turbidity currents are common in both natural (e.g., rivers) and man-made hydraulic systems (e.g., release of wastewater into a channel). Hence, understanding the characteristics and dynamics of turbidity currents is of great interest for engineers and scientists^4,5^.

Release of a dense fluid into a lighter ambient fluid from a non-continues source (e.g., the lock-exchange technique) or a continuous source (e.g., a dense jet) has been studied in several experimental investigations^6–18^. Experimental investigations also assessed the interactions between turbidity currents of different densities and velocities with an obstacle of varying geometrical features including height and width^19–27^. Alexander et al. (1994) experimentally investigated the effects of the bed topography on the flow and accumulation of the sediment, by studying variations in the depth of the dense flow and velocity before reaching the obstacle^19^. The impacts of the obstacle’s height on the blockage of the dense flow was studied by Woods et al. (1998). They concluded that an increase in the obstacle height can result in flow obstruction^28^. Morris et al. (2003) experimentally showed the influence of obstacles on the increase of thickness of the sedimentation layer at a considerable distance upstream of the obstacles^29^. Kubo (2004) explored the relationships between topographic features of a channel and particle deposition on ramps and humps in a series of experiments and numerical studies, concluding an increase in particles deposition downstream of the downslope and on the upslope of the humps^30^. Oshaghi et al. (2013) demonstrated that the obstacle height and the upstream velocity of turbidity currents are inversely related^19^. Khavasi et al. (2012 and 2019) studied the effects of particle size, bed slope and inlet Froude number on the stability of turbidity currents. They demonstrated that an increase in the particles size, bed slope and inlet Froude number can diminish the stability of the dense flow regime^31–34^.

Numerical approaches have also been used to study the dynamics of turbidity currents. Toniolo et al. (2007) developed a numerical framework to predict the trapping efficiency of turbidity currents in reservoirs and showed the impacts of topology on the reduction of fine particles settling efficiency^35^. Oehy et al. (2007) compared solid and porous obstacles confronting turbidity currents and showed a slight reduction in the trapping efficiency for porous obstacles^36^.

Several turbulence models and simulation approaches have been used to study turbidity currents including Direct Numerical Simulation (DNS), Large Eddy Simulation (LES) and Reynolds-averaged Navier–Stokes equations (RANS). RANS turbulence models are less computationally expensive in comparison with LES and DNS. RANS models with k-ε turbulence closure have been used in several studies of turbidity currents^37–41^. However, RANS models usually fail to accurately resolve flow zones with intense shear (near walls or obstacles) and flow of low to moderate Reynolds number^42,43^. Additionally, the numerical constants in RANS models need careful tuning procedures for the specific flow conditions in order to improve the accuracy of the solution, given that the Reynolds stresses in the RANS equations depend on the boundary and flow conditions. In the LES models, filtered-out eddies are not influenced by the flow conditions as the governing equations are derived based on the physical properties of the flow^37,44^. This study, for the first time, develops a numerical simulation framework using an LES turbulence model to investigate turbidity currents confronting obstacles of various geometrical configurations in a narrow channel. Also, for the first time, this study investigates the effects of discretization of particles size distribution on sediment deposition and propagation in narrow channels. The flow hydrodynamics and sediment concentration of turbidity currents over two obstacles of varying geometrical configurations in a narrow channel are investigated using the validated method described in this paper. This study highlights the capabilities of the LES numerical approaches for robust and accurate prediction of turbidity currents.

## The model

**Figure 1:**
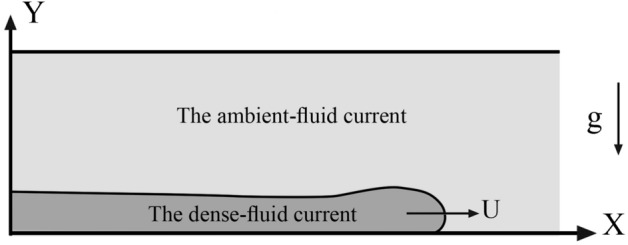
Schematic description of a dense current problem.

A dense current occurs when a dense fluid propagates into a lighter fluid. The dense current is propagated under the combined influence of its initial momentum and the gravity body force^1^ (Fig. 1). A lab-scale narrow channel containing freshwater is considered to investigate the behavior of turbidity currents. The choice of the narrow channel in this study is to characterize the augmented effects of the shear stress caused by side walls. A dense current is released into the channel and the interactions of the dense current with the fresh water (ambient) flow is simulated using LES model described in below.

Fluid flow is governed by the Navier–Stokes equations including continuity and momentum conservation equations. The concentration of particles in the dense current is modelled with a transport equation. The density difference between dense and light (ambient) fluids is assumed to be sufficiently low so that the Boussinesq approximation remains valid for modelling buoyancy forces. The gravity-buoyancy term in the momentum equation is defined as^45^:

1 $$\left(\rho -{\rho }_{0}\right)g={\rho }_{0}\beta \left(C-{C}_{0}\right)g$$

where $C$ and ${C}_{0}$ represent the normalized particle concentrations [unit less] at density $\rho$ and ${\rho }_{0}$ [kg/m^3^] for the dense and ambient fluid, respectively. The volumetric coefficient of expansion for the particles is $\beta =1$ [dimensionless] and $g$ represents the gravitational acceleration [m/s^2^]. In this study,$C$ varies from ${C}_{0}=0$ to ${C}_{max}=1$. To conduct a Large Eddy Simulation (LES), the continuity and momentum conservation equations are defined as:

2 $$\frac{\partial \overline{{U}_{i}}}{\partial {x}_{i}}=0$$

3 $$\frac{\partial \overline{{U}_{i}}}{\partial t}+\frac{\partial \overline{{U}_{i}{U}_{j}}}{\partial {x}_{i}}=-\frac{\partial \overline{p}}{\partial {x}_{i}}+\frac{\partial }{\partial {x}_{j}}\left(\frac{1}{{Re}_{b}}\frac{\partial \overline{{U}_{i}}}{\partial {x}_{j}}\right)-\frac{\partial {\tau }_{ij}}{\partial {x}_{j}}+\overline{C}{e}_{i}^{g}$$

where $\bar{U}$ represents the filtered velocity [m/s], $t$ is the time [s], ${e}_{i}^{g}$ denotes the unit vector pointing in the direction of gravity and $\bar{p}$ is the filtered pressure [kg/m s^2^]^36,46–50^. Reynolds number in this system ${Re}_{b}$ is determined based on the buoyancy velocity (U_b_):

4 $${U}_{b}=\sqrt{{H}_{inlet}\times g\frac{\rho -{\rho }_{0}}{{\rho }_{0}}}$$

5 $${Re}_{b}=\left(\frac{{U}_{b}{H}_{inlet}}{\nu }\right)$$

where $\nu$ is the kinematic viscosity [m^2^/s] and ${H}_{inlet}$ denotes the height of dense fluid at the inlet [m]. The ratio of the kinematic viscosity $\nu$ to the diffusion coefficient of suspended particles $D$ [m^2^/s] is known as the Schmidt number (Sc). The length scales are computed by the Batchelor scale ${\lambda }_{B}$ [m] showing the smallest scale for a diffusing scalar. The ${\lambda }_{B}$ is defined as the ratio of the Kolmogorov length scale $\eta$ [m] to the square root of the Schmidt number $Sc$ as^44^:

6 $$\eta = \left( {\frac{{\upsilon ^{3} }}{\varepsilon }} \right)^{{1/4}}$$

7 $${\lambda }_{B}=\frac{\eta }{\sqrt{Sc}}$$

where $\varepsilon$ is the local dissipation rate of the turbulent kinetic energy [m^2^/s^3^]. In order to simulate the smallest diffusive scales, high resolution mesh is required, which significantly increases the computational costs. The previous studies have reported that $Sc\ge 1$ has no remarkable effects on the computational accuracy^44,49,51–54^. In this study, to maintain reasonable computational costs, the Schmidt number is assumed to be $Sc=1$.

The particles inertia forces and particle–particle interactions are not computed as the concentration of suspended solids is relatively low^52,55^. Therefore, particle’s transport is simultaneously governed by the flow hydrodynamic and Stokes’ settling velocity^56^:

8 $${U}_{s}=\frac{{d}_{p}^{2}\left({\rho }_{p}-\rho \right)g}{18\mu }$$

where $\mu$ is the dynamic viscosity [kg/m.s], ${d}_{p}$ denotes the diameter of particles and ${\rho }_{p}$ is the density of particles [kg/m^3^]. Ten different particle size intervals ranging from 0.5–100 μm are considered to represent the particles with an identical density ${\rho }_{p}$. For each particle size, the Eulerian continuum transport equation is implemented according to Eq. (9)^57^, with a constant value for the Stokes’ settling velocity ${U}_{s}$:

9 $$\frac{\partial \overline{{C}_{i}}}{\partial t}+\frac{\partial \overline{{(U}_{i}+{U}_{s}){C}_{i}}}{\partial {x}_{i}}=\frac{\partial }{\partial {x}_{i}}\left(\frac{1}{Sc{Re}_{b}}\frac{\partial \overline{{C}_{i}}}{\partial {x}_{i}}\right)-\frac{\partial {\tau }_{i}^{C}}{\partial {x}_{i}}$$

Effects of the filtered fluctuations of the flow hydrodynamic are considered by the momentum and concentration residual-stress tensors ${\tau }_{ij}$ and ${\tau }_{i}^{C}$:

10 $${\tau }_{ij}=\overline{{U}_{i}\space {U}_{j}}-\overline{{U}_{i}} \space \overline{{U}_{j}}$$

11 $${\tau }_{i}^{C}=\overline{{C\space U}_{i}}-\overline{C} \space \overline{{U}_{i}}$$

**Figure 2:**
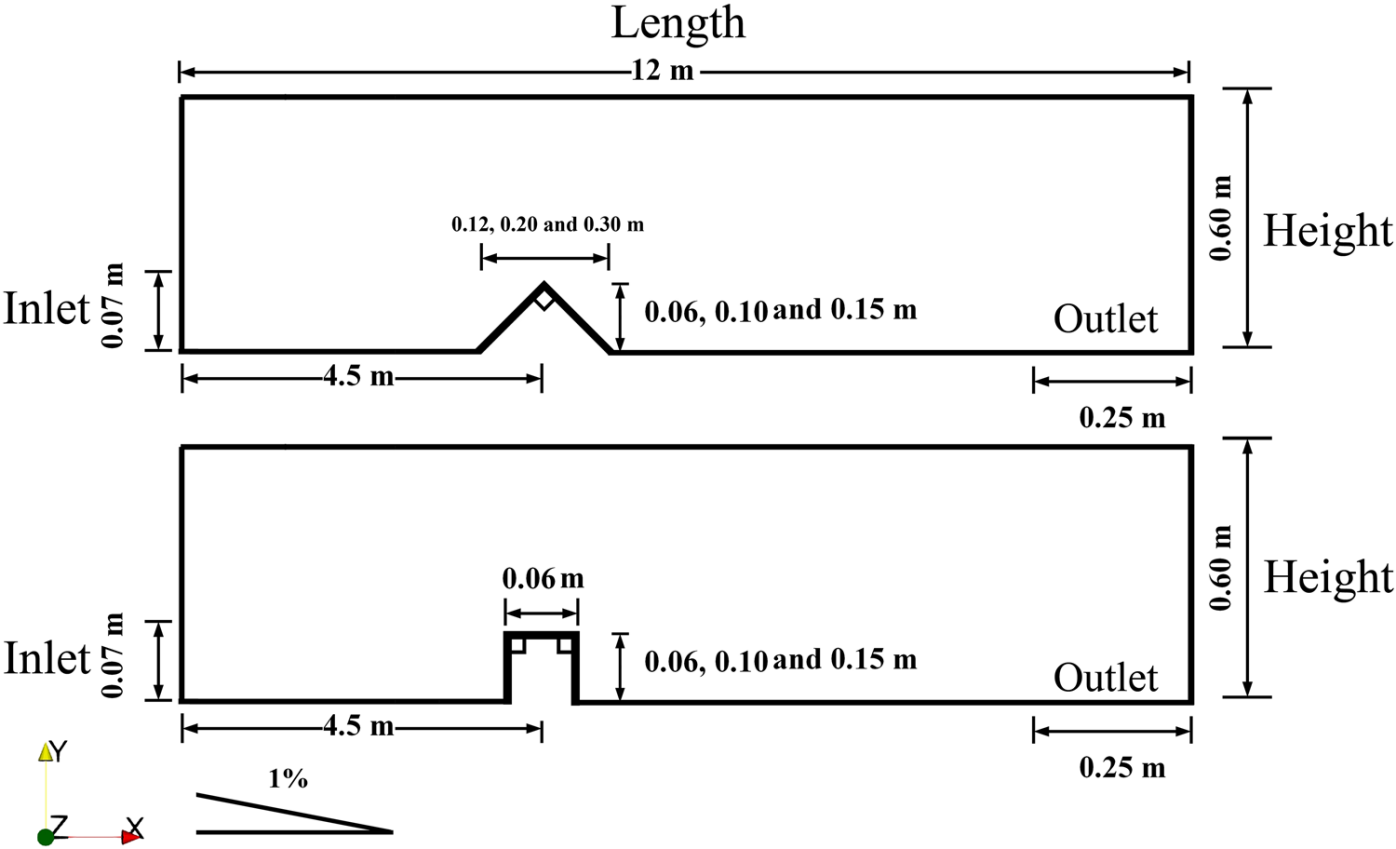
Schematic of the numerical domain and channel geometry with rectangular and triangular obstacles (subfigures are not drawn to scale).

Turbulence effects are modelled using Smagorinsky closure model^58^, including a SGS eddy-viscosity ${\upsilon }_{SGS}$ model to compute the residual tensors:

12 $${\tau }_{ij}=-2{\upsilon }_{SGS}\overline{{S}_{ij}}$$

13 $${\upsilon }_{SGS}={\left({C}_{s}\Delta \right)}^{2}\sqrt{\overline{{S}_{ij}{S}_{ij}}}$$

14 $$\overline{{S}_{ij}}=\frac{1}{2}\left(\frac{\partial \overline{{U}_{i}}}{\partial \overline{{x}_{j}}}+\frac{\partial \overline{{U}_{j}}}{\partial \overline{{x}_{i}}}\right)$$

where $\overline {{S}_{ij}}$ is the strain tensor, Smagorinsky coefficient is taken as ${C}_{s}=0.2$, $\Delta$ denotes the filtered width and turbulent Schmidt number (which is from the order of unity^59,60^) is taken as 1. The computational domain is set up in a three-dimensional Cartesian coordinate system including rectangular channel 12 m long, 0.2 m wide and 0.6 m deep, based on Farizan et al. (2018) experimental investigations^61^. The numerical flume is then utilized to investigate the hydrodynamic behaviour of turbidity current on a sloping bed subjected to different obstacles configurations. The channel bed is assumed to be smooth with a slope of 1% (Fig. 2). A continuous dense current introduced into the channel, with a constant particle density $\rho =2649 \left(kg/{m}^{3}\right)$ and a mean diameter ${D}_{50}=11 \mu m$.

Two obstacle configurations with triangular and rectangular geometries were tested in this study. The effects of obstacle’s height on the turbidity current were investigated by three different obstacle’s height of 0.06 m, 0.10 m and 0.15 m. The obstacles were located 4.5 m downstream of the inlet to minimize effects of the inlet boundaries on the particles settling rate. The inlet flow densimetric Froude number Eq. (15) was set to 0.8 for all simulation cases, ensuring a sub-critical flow condition.

15 $${F}_{inlet}=\frac{{U}_{0}}{\sqrt{g{^{\prime}}{H}_{inlet}cos\theta }}$$

where ${U}_{0}$ is the mean velocity of the turbidity current at the inlet, ${H}_{inlet}$ is the height of the inlet and the bottom slope is $\theta$. The reduced gravity acceleration is determined as^61^:

16 $${g}^{^{\prime}}=\frac{g(\rho -{\rho }_{0})}{{\rho }_{0}}$$

where *ρ* and ${\rho }_{0}$ are density of the turbidity current and the ambient fluid, respectively.

Six simulation scenarios are designed to determine the effects of obstacles height and geometrical shape on the behavior of turbidity currents. A summary of simulation cases is shown in Table 1.

**Table 1:** Summary of simulation scenarios.

Case	Inlet densimetric Froude number	Inlet concentration (kg/m^3^)	Height of the obstacle (cm)	Type of obstacle
1	0.8	6.75	6	Triangle
2	0.8	6.75	10	Triangle
3	0.8	6.75	15	Triangle
4	0.8	6.75	6	Rectangle
5	0.8	6.75	10	Rectangle
6	0.8	6.75	15	Rectangle

To guarantee an appropriate determination of the turbulent boundary layer, the first grid cell adjacent to the solid boundary is resided in the viscous sub-layer ${(y}^{+}<5)$ for a robust resolve of the boundary layer^50^. The location of this grid cell in plus units and associated parameters are described by Eqs. (17–20)^62,63^:

17 $$\frac{{U}_{1}}{{U}_{\tau }}={y}^{+}$$

18 $${y}^{+}=\frac{{U}_{\tau }\Delta y}{\upsilon }$$

19 $${U}_{\tau }=\sqrt{\frac{{\tau }_{w}}{\rho }}$$

20 $${\tau }_{w}=\mu \frac{\partial U}{\partial y}$$

where ${y}^{+}$ is the dimensionless distance from the wall, ${U}_{1}$ represents the velocity at the first cell, $\Delta y$ is the distance of the first cell from the solid boundary, ${\tau }_{w}$ and ${U}_{\tau }$ are wall shear-stress [kg/m⋅s^2^] and associated friction velocity, respectively^64^. The height of the first cell was determined using the well-established empirical correlations described by Eqs. (21–23)^50,62,63^:

21 $${\tau }_{w}=\frac{1}{2}{c}_{f}\rho {U}^{2}$$

22 $$c_{f} = 0.0577Re_{x}^{{-1/5}}$$

23 $${Re}_{x}=\frac{{U}_{x}}{\upsilon }$$

where ${c}_{f}$ is the wall skin friction coefficient, ${Re}_{x}$ denotes the Reynolds number based on the boundary layer thickness, ${U}_{x}$ is the inlet velocity [m/s] and ${c}_{f}$ is an empirical constant computed based on Reynolds number described by Eq. (23).

## Model verification

The numerical model was developed using finite-volume technique and computational codes were written in C++ with OpenFOAM (V6) open-source license. Second-order limited linear scheme was implemented for discretizing governing equations, except for the transport equation where a second-order QUICK scheme was adopted. The numerical robustness and accuracy of the QUICK scheme have been demonstrated by previous related studies^46–48,51^. The Pressure Implicit with Splitting of Operators (PISO) algorithm was implemented to solve the filtered LES equations in a transient mode. A two-steps corrector was considered in the PISO algorithm to guarantee computational robustness and a better convergence (i.e. pressure equation is corrected two times per time-step to satisfy the continuity equation)^65–67^. A series of numerical simulations were conducted to verify the numerical method and the developed model.

In turbidity currents, where complex phase-coupling (momentum exchange) between particles and fluid exists, velocity profiles determines the sediment deposition and the characteristics of flow hydrodynamic. A common method to evaluate the performance of numerical methods and computational codes is comparing the numerical results with physical modelling measurements^36,47,68–74^. Comparison of vertical variations of the numerical velocity profiles with the experimental measurements of Farizan et al. (2018) was conducted to validate the numerical model described in^61^.

**Figure 3:**
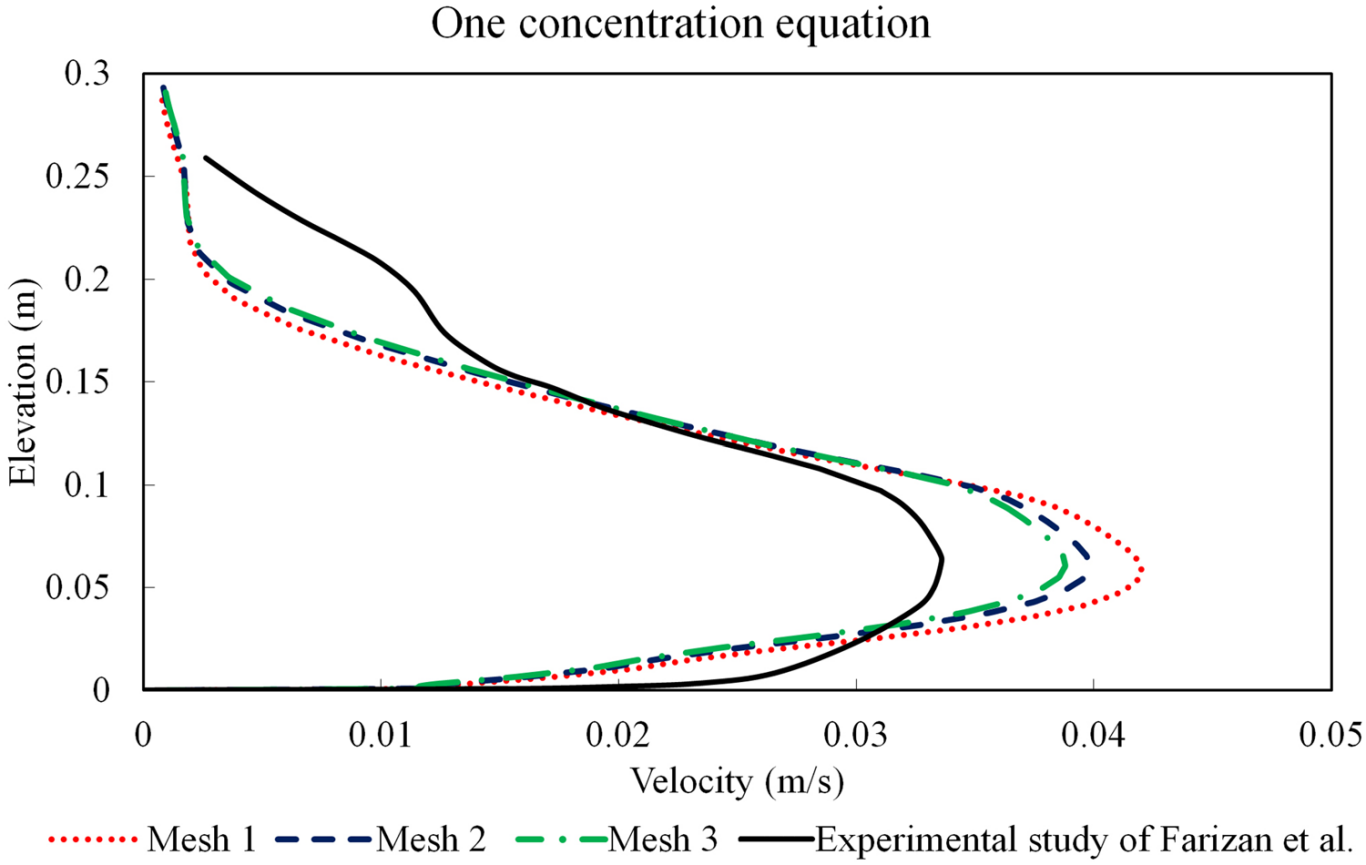
Comparison of the numerical and physical modelling of velocity profiles for the turbidity current with one concentration equation at x = 4 m (0.5 m upstream of the obstacle) for triangular obstacle (case 1).

The temporally-averaged velocity profiles at 0.5 m before the obstacle were obtained once the steady-state condition is reached, and then are compared with the experimental measurements of Farizan et al.^61^. For the validation case, channel (L:12 m, W: 0.2 m and H: 0.6 m) with a triangle obstacle located at 4.5 m away from the inlet was considered. The inlet geometry has the same width as the channel (= 0.2 m) with the height of 0.07 m. A fully developed flow condition is applied at the inlet for the turbidity current entering into the channel to include turbulent flow fluctuations. Three grid resolutions with 3.900, 4.350 and 5.590 million structured hexahedron cells (namely Mesh 1, Mesh 2 and Mesh 3, respectively) were considered along with a mean particle diameter of ${D}_{50}=11 \mu m$, to conduct sensitivity analysis and mesh dependency study. Time-averaged velocity profiles at a distance of 4 m from the inlet were determined for the validation test cases (Mesh 1, Mesh 2 and Mesh 3) with one concentration transport equation, and compared with the laboratory measurements of Farizan et al.^61^ (Fig. 3). The comparison of the results presented in Fig. 3 highlights considerable deviations between experimental and numerical velocity profiles. The numerical model with one concentration transport equation overestimates the shear effects in the dense current and the velocity profile near the bed (the lower part of the velocity profile). It was shown that by ignoring the particles larger than ${D}_{50}$, the velocity of turbidity current near the bed was increased.

**Figure 4:**
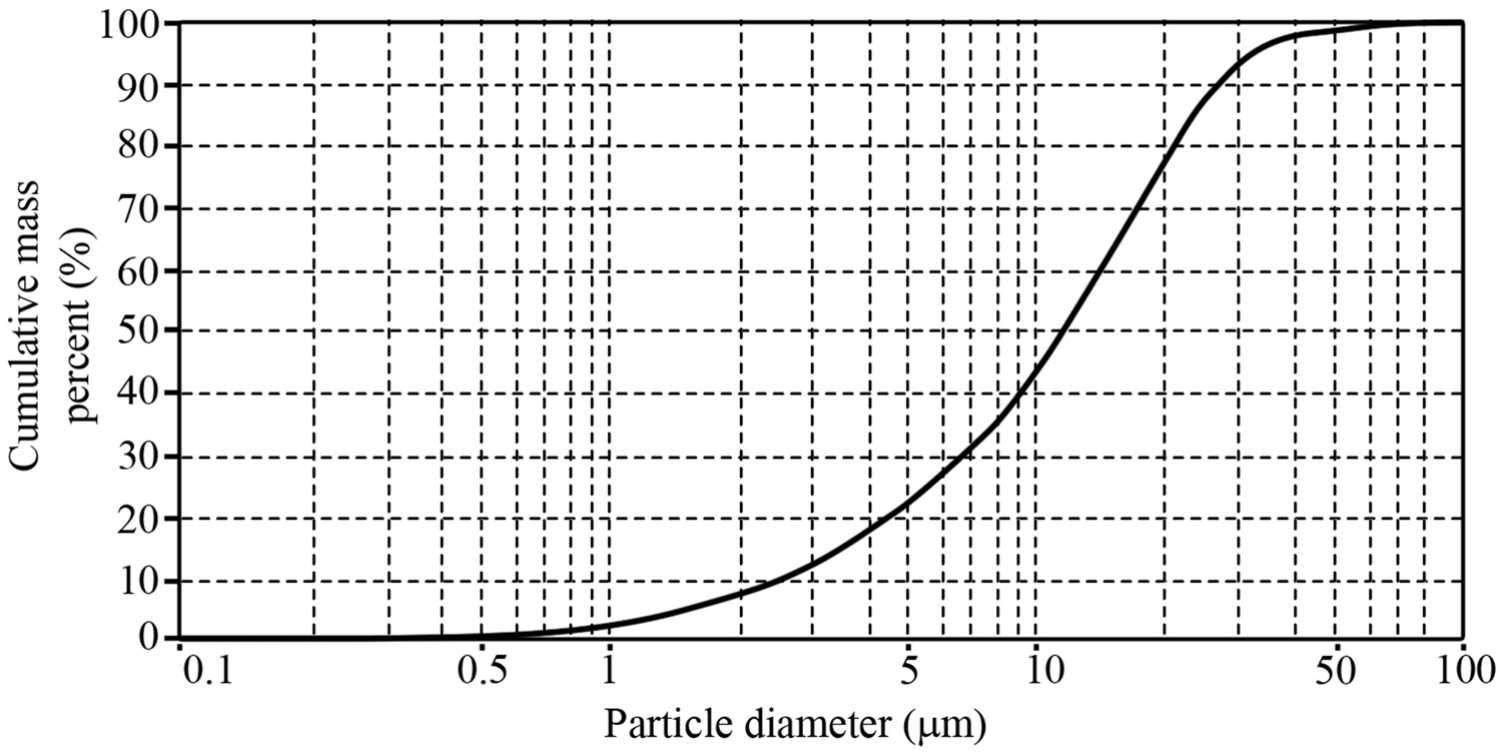
Particle diameter distribution in this study. Adopted from Farizan et al. (2018)^61^.

To improve the discrepancy between numerical results and the measurements, additional concentration transport equations were considered. Additional simulation cases with two, five and ten concentration transport equations were conducted to produce a more robust estimation of the particles size and distribution (0.5–100 μm). Figure 4 shows the distribution of particles experimentally measured by Farizan et al. (2018)^61^.

**Figure 5:**
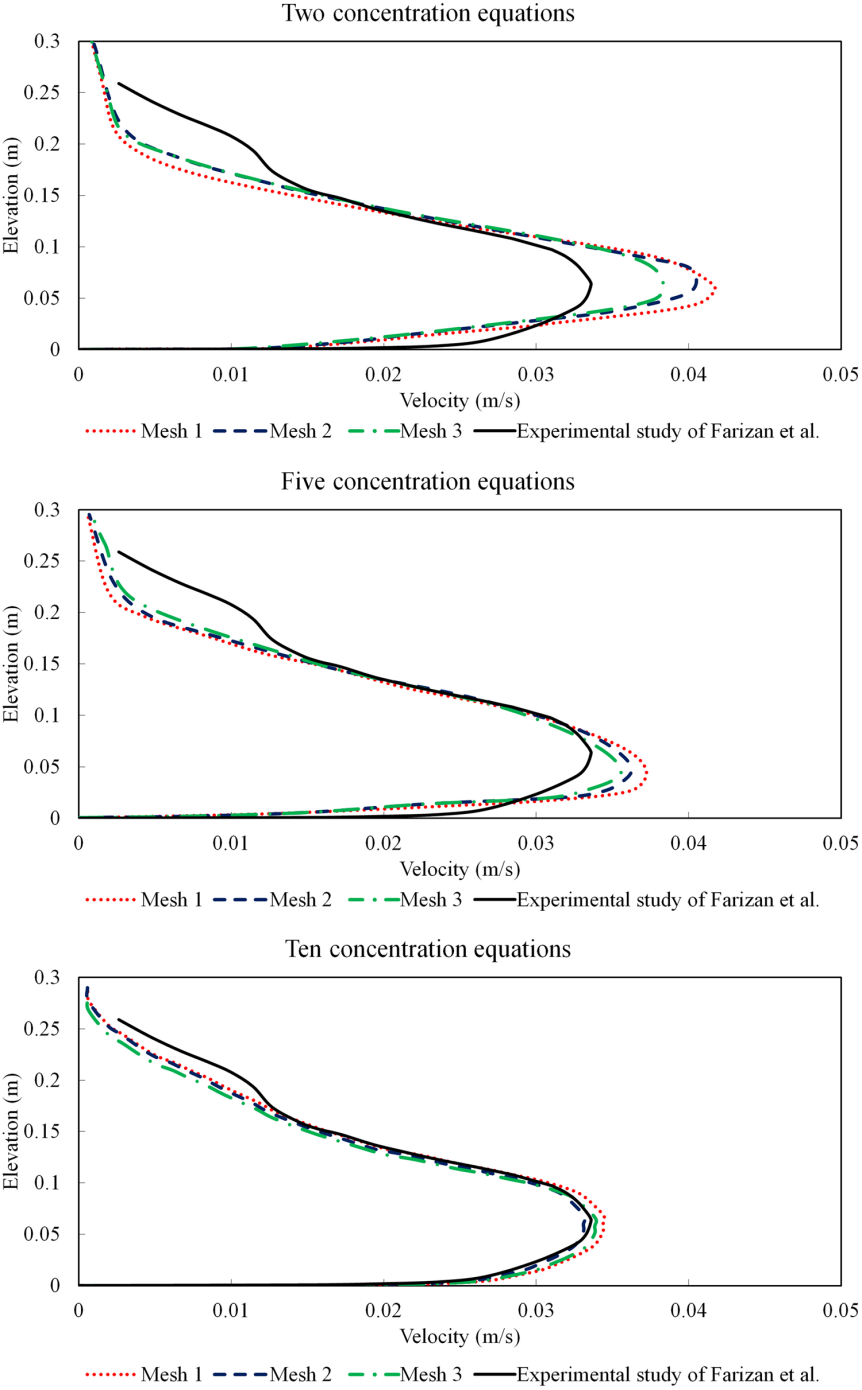
Comparison of the numerical and physical modelling of velocity profiles for the turbidity current with two, five and ten concentration equations at x = 4 m (0.5 m upstream of the obstacle) for triangular obstacle (Case 1).

**Figure 6:**
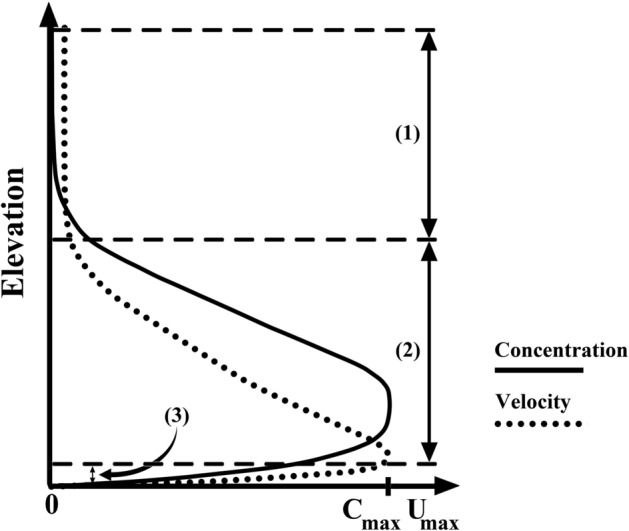
Schematic of the velocity and concentration profiles of turbidity currents^31^.

Figure 5 compares the velocity profiles from numerical simulations with the experimental measurements of Farizan et al. (2018)^61^. The simulation sets presented in Fig. 5 include extra particle size intervals and more transport equations to enhance the computational accuracy of the velocity profiles. The simulations show that for all the cases, the velocity in the upper region of the turbidity current (see Fig. 6) is increased as finer particles are introduced to the flow, while the lower region of the turbidity current (near the channel bed) is slowed down due to the effects of larger particles. The accuracy of the numerical results is improved by increasing the number of concentration transport equations and the best performance was achieved for the case of 10 concentration transport equations. Following grid dependency analysis, to achieve numerical stability, computational accuracy and cost-effective solution, Mesh 2 with 4.350 million structured hexahedron cells was selected for further simulations.

Despite the shear stress effects of walls on the fluid might initially seem to be significant, in this narrow channel, the results showed that at the Reynolds number considered in this study the walls effect is negligible, which is in good agreement with the study by Khavasi et al. and Oehy et al.^31,32,36,68^.

## Results and discussion

The velocity and concentration profiles inside the dense layer of the turbidity current are categorized into three distinct regions (Fig. 6): (1) the upper part is known as a shear layer region where the density of the turbidity current decreases and asymptotes to the counterpart value for the ambient fluid; (2) the middle part is known as the suspension zone where the majority of particles are suspended in the fluid; and (3) the lower zone which is a depositional area where particles are settled^31^. In this paper, the upper zone of the channel (part 1 in Fig. 6) is not described as the flow velocity asymptotes to zero^61^.

Velocity and concentration values are measured after reaching a quasi steady-state to avoid temporal fluctuations in the flow parameters.

Sediment deposition and entrainment rates are computed by measuring the vertical sediment flux and variations in the concentration of particles along the length of the channel. Suspended sediment flux per unit width is determined using Eq. (24) as:

24 $${q}_{s}=\sum_{0}^{{z}_{upper}}u\left(z\right)c(z)\Delta z (4)$$

where ${z}_{upper}$ is the upper boundary over which the concentration becomes negligible, $u\left(z\right)$ is the sum of settling and horizontal velocity and $c(z)$ is the concentration for the dense current.

**Figure 7:**
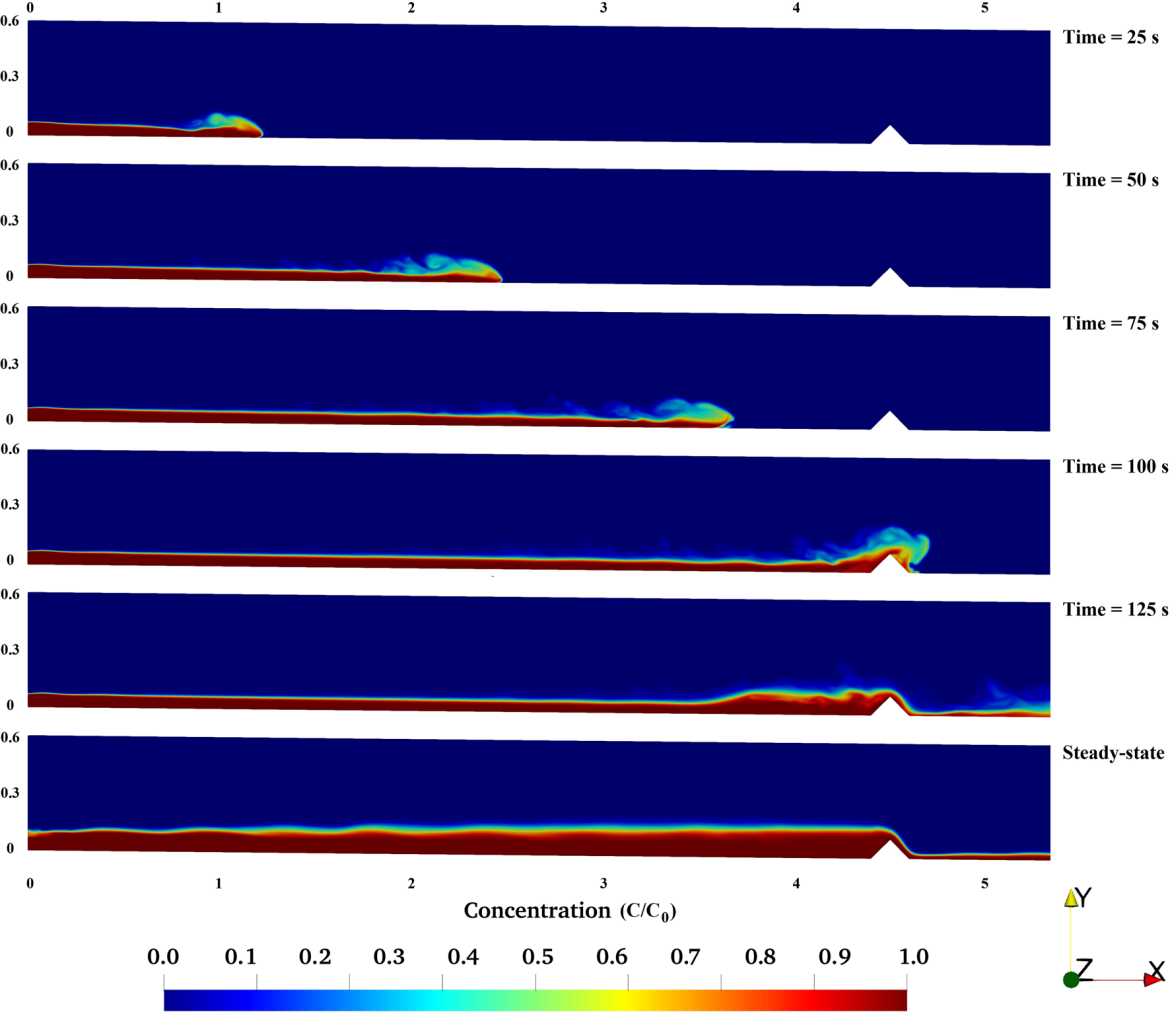
Simulation results for turbidity current flowing over triangular obstacle.

**Figure 8:**
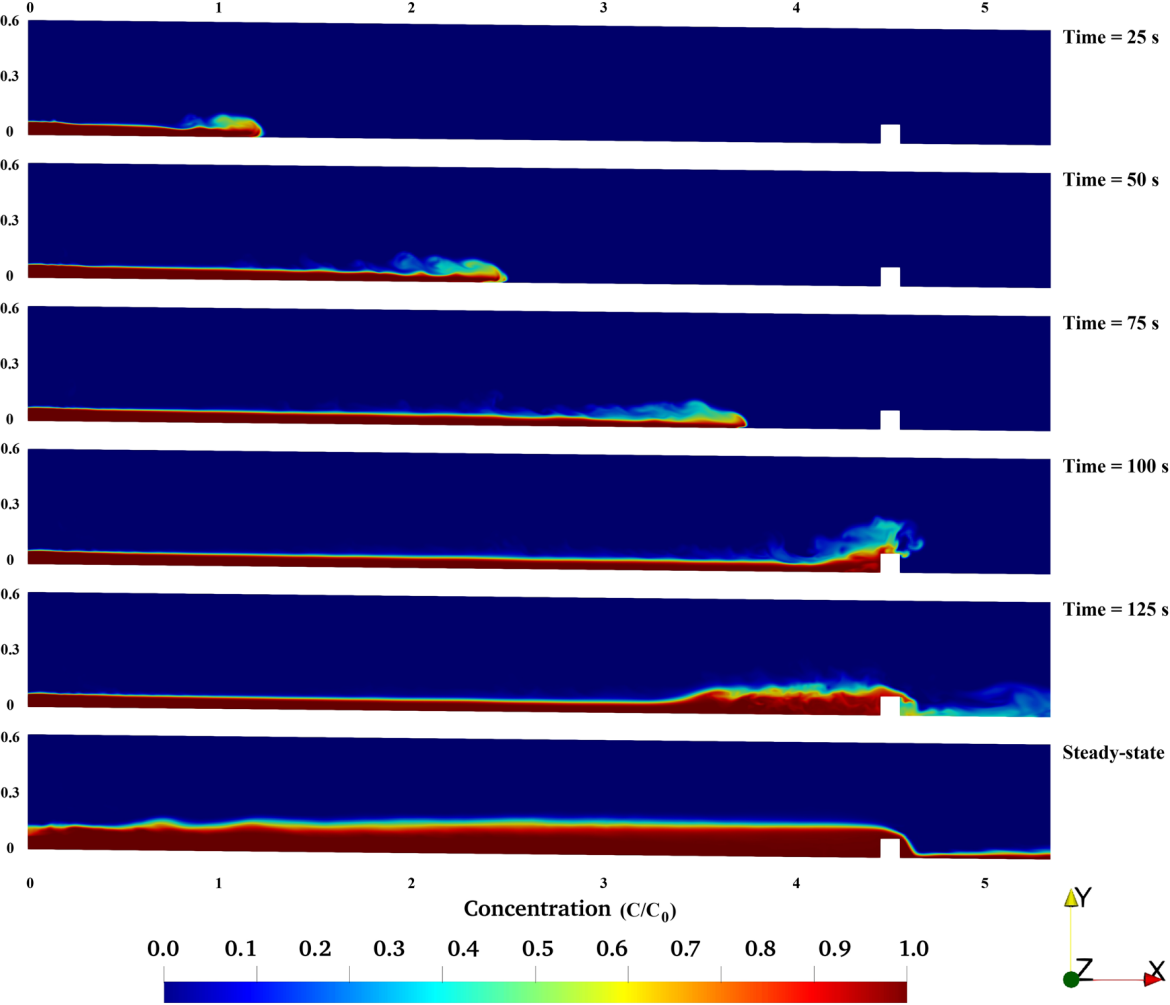
Simulation results for turbidity current flowing over rectangular obstacle.

The turbidity bore is defined as a moving hydraulic jump over the bed of the channel. Obstacles alter the flow regime and can move the internal bores towards the inlet of the channel. Also, the flow hydrodynamic characteristics significantly impact the position and structure of turbidity currents on the channel bed^36,75^. Figures 7 and 8 show temporal evolution of the turbidity current in the channel, indicating multiple reflected bores of the turbidity current as it travels inside the channel and over the obstacle. The horizontal velocity of turbidity current slightly decreases when the flow climbs up the obstacle which is due to the flow-obstacle interactions and the consequent dissipation of turbulent kinetic energy of the current (Figs. 7, 8). The bore of the dense flow becomes thinner immediately after it passes the obstacle and as it moves towards the outlet. For the case of both obstacles (Figs. 7, 8) sediment deposition and formation of an interface between turbidity current and the fluid of lighter density is observed^36^.

**Table 2:** Sediment flux variation Eq. (25).

X (m)	H = 6 cm (%)	H = 10 cm (%)	H = 15 cm (%)
4	3.38	4.76	10.1
4.5	1.14	1.54	3.47
5	0.98	1.02	2.34
5.5	0	0.98	1.57
6	0	0.4	0.97
6.5	0	0	0
7	0	0	0
7.5	0	0	0

Simulations were continued until the sediment deposition behind the obstacle reached a steady height. Temporally-averaged flow characteristics were determined once the quasi steady-state condition is met, to avoid temporal fluctuations of the LES.

The comparison between Figs. 7 and 8 at *t* = 125 s demonstrates an intensified turbulent hydraulic jump when the turbidity current passes over the rectangular obstacle. However, the flow over the triangular obstacle can be characterize as a quasi-uniform jump with a less disturbed flow.

Turbidity currents can significantly be influenced by the height of the obstacle. Previous studies show the effects of the obstacle on blockage and reflection of turbidity currents^36,61^. The obstacle’s height of equal to twice the height of the current, was reported to cause a considerable reflection in the turbidity current flow^76^.

**Figure 9:**
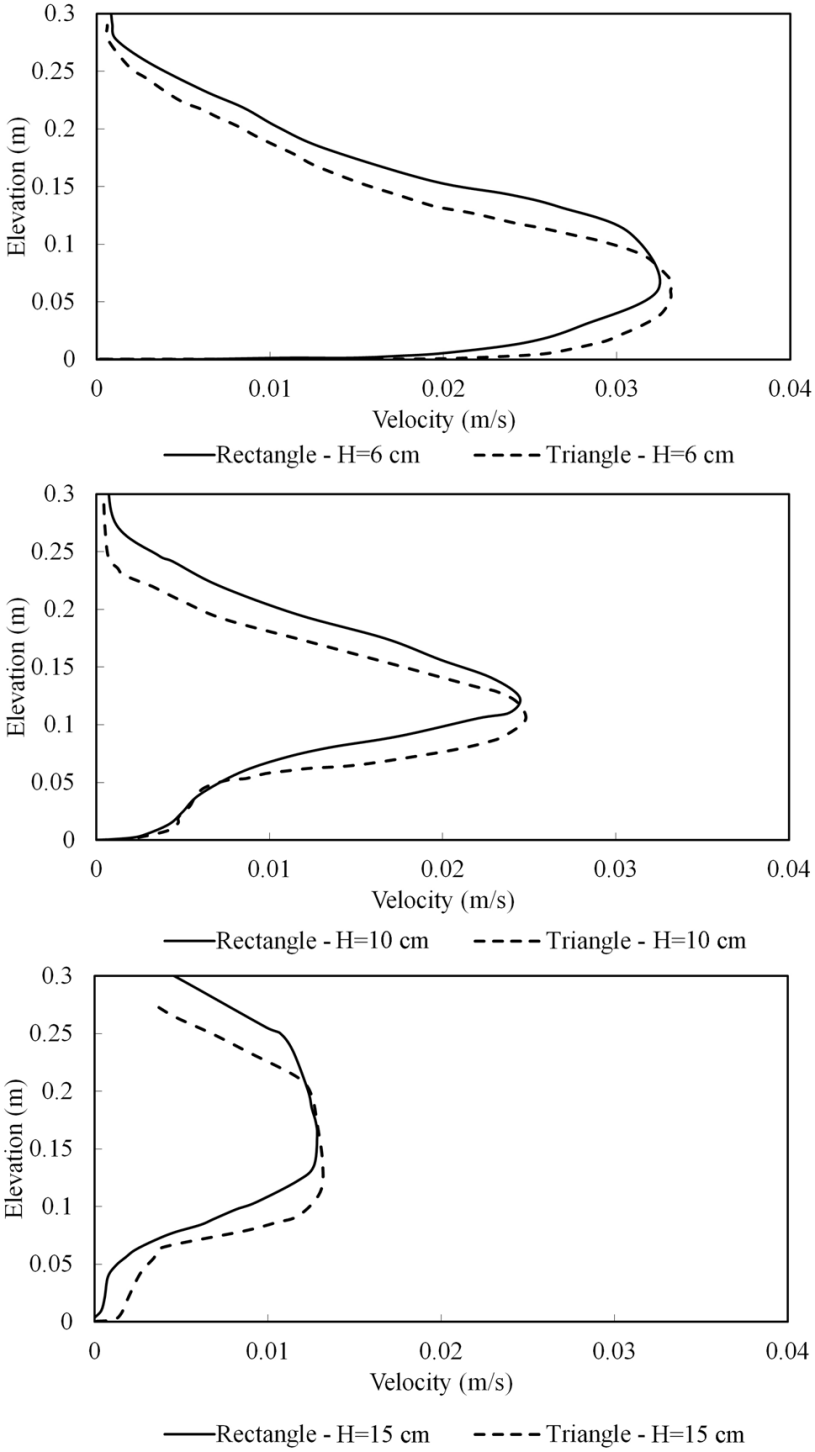
Velocity profiles of the turbidity current at x = 4 m for triangular and rectangular obstacles.

Considering different heights, the stream-wise time-averaged velocity profiles of turbidity current in the quasi steady-state were plotted at 0.5 m upstream of both rectangular and triangular obstacles (Fig. 9). The increase in the height of the obstacles considerably changed the vertical structure of the dense current’s velocity profiles. For the case of obstacle height of 0.06 m and for both geometries, the maximum velocity was observed in the settling zone behind the obstacle. For the cases with the obstacle height of 0.10 m, the velocity profile shows a sharp increase from the bottom up to the depth of 0.10 m, then a sharp reduction is seen up to the interface between the dense and ambient fluid. For the cases with the obstacle height of 0.15 m, the velocity profiles and vertical distribution of shear effects have smaller values with a less distributed pattern in comparison to the cases with smaller obstacle height. The patterns of the velocity profiles for both obstacles geometries are very similar, with the maximum velocities for the rectangular obstacle occurring at slightly higher depths from the bed. Rapid changes in the velocity profile of the turbidity current for the rectangular obstacle begin in higher elevation in contrast with triangular obstacle, which is due to the higher sediment decomposition for rectangular obstacle case.

**Figure 10:**
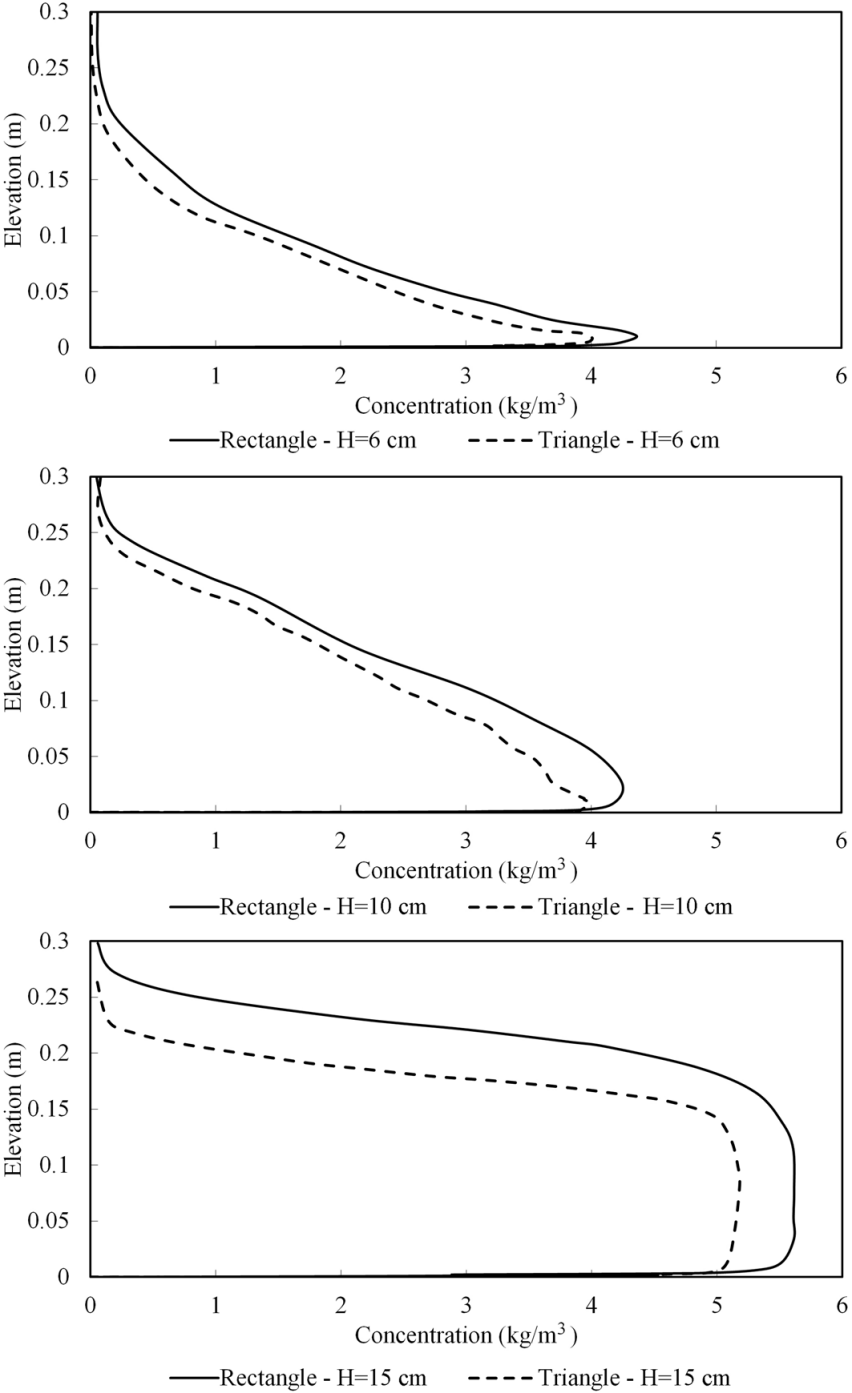
Concentration profiles of the turbidity current at x = 4 m for triangular and rectangular obstacles.

Figure 10 shows the suspended sediment concentration inside the turbidity current upstream of the obstacle at 4 m from the inlet. The thickness and average concentration of the turbidity current upstream of the obstacle is increased with the increase in the height of the obstacles. The figure shows a thicker cloud of deposition for turbidity current behind the rectangular obstacle in comparison with the triangular obstacle, highlighting the effects of obstacle’s geometrical shape on the control and mitigation of turbidity currents.

**Figure 11:**
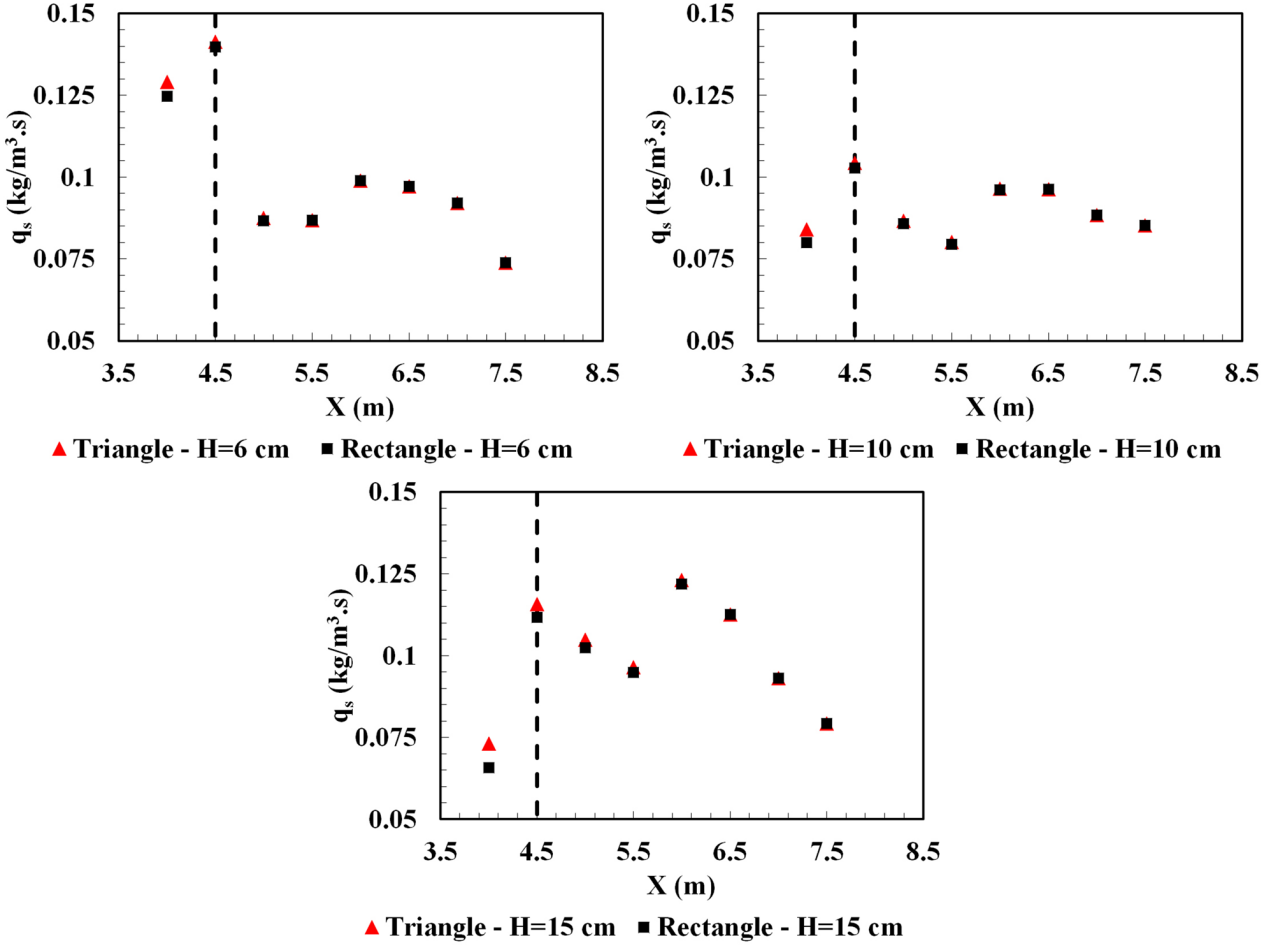
Sediment flux per unit width along the channel for all the simulation cases (dash lines show the location of obstacle).

Figure 11 shows the suspended sediments flux per unit width of the channel for all the simulation cases. Implementation of the obstacles improved the settling efficiency for the turbidity current upstream of the obstacles. Increasing the height of the obstacles slowed down the vertical variation of horizontal velocity profile which led to a reduction in the sediment flux. The presence of obstacle, regardless of its geometrical shape, had no considerable impact on the settling rate at the downstream of the channel. The settling deposition of particles for the cases with 0.10 m and 0.15 m obstacles in the upstream of the obstacles are almost equal.

The effects of the obstacle’s geometrical shape on the sediment deposition was more pronounced for the rectangular obstacles, mainly due to the higher reduction in the dense flow velocity behind the rectangular obstacle. In order to compare the obstacles shape impact, the difference in sedimentation flux-rate $({q}_{s})$ for triangular and rectangular obstacles are determined (Table 2). Positive values indicate a dominancy in the settling of suspended sediments for the rectangular obstacle. Accordingly, rectangular obstacles are suggested to be implemented in channels leading to hydraulic control structures. More deposition of the sediments increases the efficiency of hydraulic structures in water systems^44^.

25 $${q}_{s\_variation}=(\frac{{q}_{s (triangular obstacle)}-{q}_{s (rectangular obstacle)}}{{q}_{s (triangular obstacle)}})\times 100$$

## Conclusion

Appropriate understanding and analysis of turbidity currents are vital for sustainable and efficient management and operation of natural and man-made hydraulic structures. This study develops and successfully validates a high-resolution numerical simulation model using mathematical capabilities of Large Eddy Simulation (LES) technique. The effects of the number of concentration transport equations on the robustness and numerical accuracy were studied in detail. The results highlight that discretization of the particles size distribution improves the accuracy of the model in predicting turbidity current hydrodynamics and spatiotemporal structure of turbulence.

The effects of obstacle’s geometrical shape and height on the turbidity currents characteristics in a narrow three-dimensional channel were modelled. Two obstacle prototypes of rectangular and triangular shape with varying height were investigated. The numerical velocity and concentration profiles were determined for all simulation cases described in Table1.

The findings indicate that, for both rectangular and triangular obstacles, by increasing the height of the obstacle, the maximum velocity of the turbidity current was reduced and the shape of the vertical distribution of flow hydrodynamic in the dense layer was changed. The results show that the increased height of obstacle directly impacted the vertical structure of shear and turbulent velocity. Across all test cases, comparison between the two obstacle geometries shows that for both obstacles the overall shapes of flow hydrodynamics are similar.

Furthermore, a direct relationship between the obstacle’s height and the settling capability of the obstacles was observed. The numerical results highlight that the shape and height of obstacles significantly change the hydrodynamics, sediment particle distributions and structure of turbidity currents over a smooth bed channel. Installation of a rectangular obstacle is recommended to enhance the deposition and efficiency of hydraulic structures (dams, reservoirs and weirs) in water systems.

The computational framework developed in this study demonstrates that LES modelling can be implemented as computationally robust and reliable numerical technique to investigate the dynamics of turbidity currents in turbulent flow conditions.

## Data Availability

The numerical model is developed in OpenFOAM (v6) under open-source license. All the simulation and data analysis codes are developed in C++ and can be made available by request from the corresponding author.
